# Effects of mindfulness-based digital health interventions on BMI and weight-related behaviours in adults: a systematic review and meta-analysis

**DOI:** 10.3389/fnut.2026.1874656

**Published:** 2026-07-28

**Authors:** Youdi Wei, Yujie Liu, Yuling Yuan

**Affiliations:** 1 School of Physical Education, Nanchang University, Nanchang, China; 2 Division of Sports Science and Physical Education, Tsinghua University, Beijing, China

**Keywords:** adults, BMI, digital health, mindfulness, weight-related behaviours

## Abstract

**Background:**

The rising prevalence of overweight and obesity has become a critical global health issue. Previous researches have demonstrated that mindfulness-based interventions can improve both BMI and weight-related behaviour. Digital delivery improves accessibility, but evidence for BMI and eating outcomes remains inconclusive. This systematic review and meta-analysis synthesizes the available evidence on the impact of such interventions on BMI and weight-related behaviour in adults.

**Methods:**

A systematic literature search was performed across PubMed, Web of Science Core Collection, and Scopus. Eligible studies evaluated the effects of mindfulness-based digital health interventions on adult BMI and weight-related behaviour (e.g., binge eating, emotional eating). Data extraction and risk of bias assessment were carried out independently, and meta-analyses were conducted on pre-post intervention outcomes.

**Results:**

A total of 10 studies were included involving 902 participants. Mindfulness-based digital health interventions may significantly improve binge eating [SMD = −0.59, 95%CI: (−1.07, −0.10)], and emotional eating [SMD = −0.23, 95%CI: (−0.45, −0.02)]. No significant effects were observed for uncontrolled eating [SMD = −0.17, 95%CI: (−0.38, 0.05)] or cognitive restraint [SMD = −0.04, 95%CI: (−0.66, 0.59)]. The impact on BMI remained non-significant [MD = −0.44, 95%CI: (−1.70, 0.82)].

**Conclusion:**

Mindfulness-based digital health interventions effectively alleviated emotional eating and binge eating; however, the improvement in BMI was not significant. These findings suggest that future research should focus on continually refining the details of the intervention protocol to enhance study robustness.

**Systematic review registration:**

https://www.crd.york.ac.uk/PROSPERO/view/CRD420251125050, PROSPERO, identifier number CRD420251125050.

## 1 Introduction

The escalating prevalence of overweight and obesity has cemented them as a pervasive global health challenge, if past patterns hold, estimates suggest that by 2050, overweight and obesity will affect over 50% of the world’s adult population (1). In non-clinical populations, maladaptive eating behaviours, such as binge eating and other problematic eating patterns, are associated with psychological distress and long-term weight gain (2, 3). The transition from general overweight to clinical eating disorders is complex, with negative affect, weight/shape preoccupation, and weight stigma consistently identified as key contributors (4). A pioneering work of Kristeller et al. (5), in the late 1990s delivered mindfulness-based interventions (MBIs) to obese women with eating behaviour issues and observed positive results, which served as a catalyst for establishing mindfulness as a novel intervention in this field.

Mindfulness is defined as the practice of directing complete attention to the experiences occurring in the present moment (6), and MBIs are interventions sharing this underlying element (7), relevant to eating behaviour is grounded in close theoretical links with emotional regulation, impulsivity, and interoceptive awareness. Specifically, mindfulness is theorized to modulate eating behaviours and weight by reducing emotional distress, enhancing self-regulatory capacity, and improving awareness of hunger and satiety signals, thereby enabling individuals to respond to internal physiological cues rather than emotional or external cues. Through these mechanisms, MBIs may improve weight-related behaviours (8, 9). In this review, “weight-related behaviour” is operationally defined as binge eating, emotional eating, uncontrolled eating, and cognitive restraint, drawing on prior literature and their consistent use as outcome measures in included studies (10).

Existing systematic reviews and meta-analyses have reported beneficial effects of traditional face-to-face MBIs on physical and psychological health outcomes in adults who are overweight or obese (11), but resource-demanding and require prolonged face-to-face engagement (12), which may be restricted by high costs, geographical limitations, stigma, and accessibility issues. Digital delivery, by contrast, offers greater efficiency and cost-effectiveness (13), which has accelerated the integration of mindfulness into digital intervention frameworks. In this review, “mindfulness-based digital health interventions” are operationalized as those MBIs delivered via internet-based platforms (E-health) (14) or mobile applications (M-health) (15). Contemporary programs development increasingly positions digital delivery mindfulness as a core module or the central component of the entire therapeutic approach, not just an add-on (10, 16), and may benefit BMI and weight-related behaviours.

As for the effect of mindfulness-based digital health interventions, previous systematic reviews have demonstrated that web-based digital health interventions can produce short-term improvements in body weight (17). A narrative review by Lyzwinski et al. evaluated the overall effectiveness of electronic MBIs on stress, weight-related behaviours, and weight loss (7), however, this review was descriptive in nature and did not provide quantitative effect estimates or objective outcome data which limits the strength of its conclusions. To build upon these prior works, this review aims to investigate the effect of mindfulness-based digital health interventions on BMI and weight-related behaviours in adults.

## 2 Methods

The protocol of the present study obtained PROSPERO registration, with the unique registration number CRD420251125050.

### 2.1 Search strategy

This study followed the Preferred Reporting Items for Systematic Reviews and Meta-Analyses (PRISMA) guidelines (18). Comprehensive searches were performed in PubMed, Web of Science Core Collection (WOS), and Scopus electronic databases up to 9 June 2026. The search terms included: (mindful OR mindfulness OR mindful eating OR mindfulness-based eating OR mindful-based intervention) AND (digital OR application OR online OR web-based OR mobile OR APP OR internet-based OR e-health OR m-health OR digital health) AND (eating behaviour OR diet behaviour OR binge eating OR emotional eating OR loss of control eating OR BMI OR body weight OR weight loss OR body mass index). Searching strategy and corresponding results of each database were shown in Supplementary Table 1. The search was confined to human research and publications in English.

### 2.2 Inclusion and exclusion criteria

To investigate the effects of mindfulness-based digital health interventions on BMI and weight-related behaviours in adults, studies meeting all of the following criteria were included: (1) Population: participants aged 18 years or older; (2) Intervention and Comparison: interventions based on mindfulness and were digital delivered (including M-health or E-health), used either alone or as part of a mixed approach incorporating digital elements; (3) Outcomes: reported BMI or weight-related behaviours; (4) Study design: randomized controlled trial (RCT). Exclusion criteria: (1) non-randomized study design; (2) reviews, commentaries, case reports, conference abstracts; (3) the control group also received a mindfulness-based digital health intervention.

### 2.3 Data extraction and synthesis

Two independent researchers were responsible for data collection and methodological quality evaluation (Y.W. and Y.L.). All emerging disagreements were adjudicated by a third senior reviewer (Y.Y.), followed by group discussion to reach consensus. Information retrieved from each study comprised the first author’s name, publication year, population characteristics, intervention details (frequency, duration, period), and outcome measures. Data from multi-study publications were extracted separately. Missing data or data presented only graphically prompted attempts to contact the original authors. Unobtainable studies were excluded from the final analysis. For each study, pre- and post-intervention mean (M), standard deviation (SD), and sample size were recorded.

### 2.4 Risk of bias assessment and assessment of methodological quality

Bias assessment was performed in six dimensions using the Cochrane Risk of Bias 2.0 (RoB 2) framework (19), which were random process, deviations from intended interventions, outcome measurement, missing outcome data, selective outcome reporting, and overall bias. Discrepancies were initially handled by discussion. If agreement remained unattainable, a third reviewer (Y.Y.) acted as the final arbitrator.

### 2.5 Data conversion

This meta-analysis was based on pre-post changes between the two groups. For each included study, we extracted group-specific means, standard deviations, and sample sizes for both the intervention and control groups at baseline and post-intervention. Pre-post M changes and SDs were calculated using these extracted data. When only confidence intervals or standard errors (SEs) were reported, we followed the Cochrane Handbook to perform the conversion (20). Since most included studies failed to report the correlation coefficients (r) between baseline and post-intervention outcomes, a fixed conservative value of *r* = 0.5 was assigned in accordance with Cochrane guidelines. All conversion equations used in this analysis are presented below (20):


$$M_{\text{change}}=M_{\text{post}}-M_{\mathrm{pre}}$$

$${\mathrm{SD}}_{\text{change}}=\sqrt{{\mathrm{SD}}_{\mathrm{pre}}^{2}+{\mathrm{SD}}_{\text{post}}^{2}-(2\times r\times {\mathrm{SD}}_{\mathrm{pre}}\times {\mathrm{SD}}_{\text{post}})}$$

Where $M_{\text{change}}$ is the raw mean difference, $M_{\mathrm{pre}}$ and $M_{\text{post}}$ are the reported pre- and post- intervention mean, respectively. ${\mathrm{SD}}_{\text{change}}$ is the SD of the difference in means, ${\mathrm{SD}}_{\mathrm{pre}}$ and ${\mathrm{SD}}_{\text{post}}$ are the SD at pre- and post-intervention, and r is the pre–post test correlation coefficient.

### 2.6 Statistical analysis

All meta-analyses were performed using R Studio software. Data synthesis was conducted when at least two comparable trials were available (21). The standardized mean difference (SMD) was classified as trivial (< 0.2), small (0.2–0.5), medium (0.5–0.8), or large (> 0.8) (22). When outcomes were reported using the same assessment method and identical measurement units across studies (e.g., BMI), the absolute mean difference (MD) was calculated instead. We adopted the *I*^2^ index and Cochrane *Q*-test to examine inter-study statistical heterogeneity. Specifically, *I*^2^ ≤ 25% indicated low heterogeneity, 25–75% indicated moderate heterogeneity, and *I*^2^ > 75% represented high heterogeneity. To address potential bias arising from multiple intervention arms, we followed established guidelines to ensure appropriate control (20). For this purpose, we divided sample size of each group by the number of comparisons in which it was involved.

Egger’s test was performed to detect potential publication bias if more than 10 studies were included. (23). When publication bias was detected (*p* < 0.05), the trim-and-fill method was adopted to evaluate its influence on the pooled outcomes (24). Sensitivity analyses were performed by excluding small-sample studies and those where mindfulness was only a minor component, to assess the robustness of the pooled results. To explore potential sources and impact indicators of heterogeneity, subgroup analyses were conducted based on intervention period according to the primary results. Only limited data were available for other dose variables. Moreover, although has suggested that MBIs may require cultural adaptation (25, 26), the limited number of studies per outcome precluded subgroup analyses by country.

### 2.7 Certainty of evidence

As recommended by Garey et al., the assessment of the overall quality of evidence should be determined by evaluating the validity of individual study outcomes (27). This meta-analysis assessed the quality of outcome evidence using the Grading of Recommendation Assessment, Development and Evaluation (GRADE) system, which rates evidence quality as “high” (further research is unlikely to alter the estimate of effect), “moderate” (further research may influence and possibly modify the estimate), “low” (further research may have an impact on the effect estimate and may change the estimate), or “very low” (any effect estimate is uncertain) (28).

## 3 Results

### 3.1 Study selection

**Figure 1:**
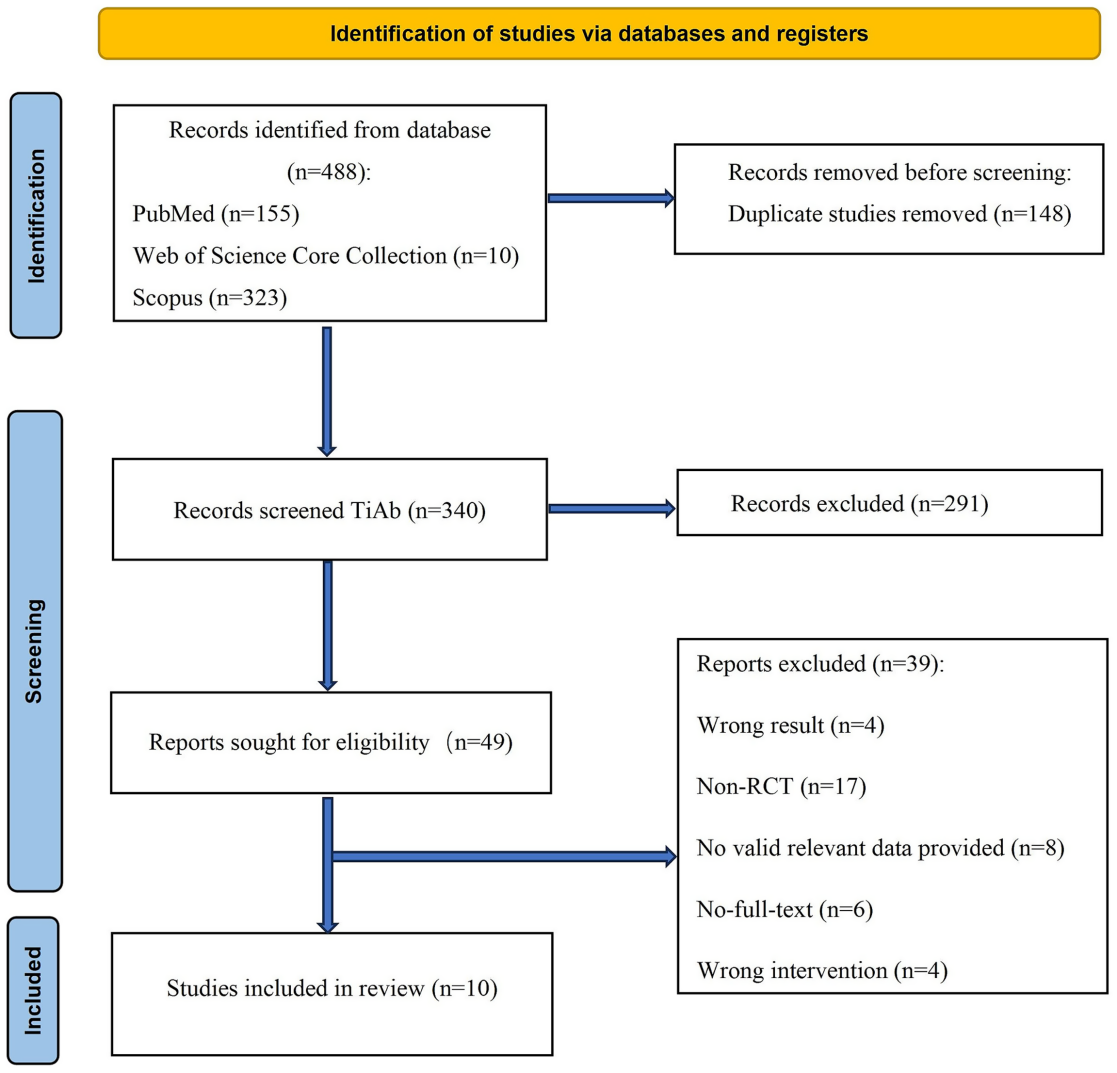
PRISMA flow chart.

A preliminary database search identified 488 publications (155 from PubMed, 10 from WOS, 323 from Scopus). After removing 148 duplicate, 340 records were screened by title and abstract, resulting in 49 reports for full-text assessment. After excluding 39 reports at the full-text review stage, ultimately, a total of 10 studies were judged as eligible for this review (Figure 1).

### 3.2 Intervention characteristics

As illustrated in Table 1, of the 10 included studies evaluated, 11 distinct interventions targeting BMI and weight-related behaviours in adults. The outcome measures varied across studies: five studies focused on BMI (16, 29–33). four studies focused on binge eating (16, 30, 33, 34), seven studies focused on eating behaviour (16, 29, 32, 35–37). Regarding digital delivery, four studies used E-health, while seven used M-health interventions.

**Table 1:** Characteristics of studies included in the review and meta-analysis.

Author	Country	Age (years)		Sample		Period	Duration	Frequency	Intervention measures		Results
		EG	CG	EG	CG				EG	CG	
Carrard et al. 2011, (29)	Switzerland	34.4 ± 11.0	37.8 ± 11.8	37	37	6 months	N/A	N/A	E-Health Audio-based exercises	Standard care	①②
Järvelä-Reijonen, et al. 2018, (35)	Finland	49.1 ± 7.7	49.2 ± 7.4	78	71	8 weeks	90 min	N/A	M-Health Video or text-based exercises	None	②
Carpenter et al. 2019, (30)	USA	45.5 ± 9.4	50.8 ± 10.5	44	22	6 months	20–30 min	N/A	M-Health Audio-based exercises	Weight Talk	③
Czepczor-Bernat et al. 2021, (36)	Poland	32.7 ± 8.3	31.0 ± 7.8	43	20	15 days	N/A	N/A	E-Health ME	None	②
Czepczor-Bernat et al. 2021, (36)	Poland	33.0 ± 6.9	31.0 ± 7.8	46	20	15 days	N/A	N/A	E-Health ME	None	②
Yu et al.2021, (16)	USA	32.0 ± 15.2	37.0 ± 18.7	8	4	3 months	N/A	N/A	M-Health Video meeting	Standard care	①②③
Radin et al. 2023, (31)	USA	38.6 ± 11.0	38.3 ± 12.6	33	33	8 weeks	10 min	5 times/week	M-Health Audio-based exercises	Standard care	①
Matsuhisa et al. 2024, (37)	Japan	69.9 ± 3.6	68.7 ± 6.3	16	14	26 weeks	8 min	7 times/week	M-Health Breathing exercise	comprehensive lifestyle intervention	②
Linardon et al. 2024, (34)	Australia	39.7 ± 12.5	38.6 ± 11.9	121	117	12 weeks	N/A	N/A	M-Health N/A	Not mindfulness-based intervention	③
Moreira et al. 2024, (32)	Brazil	35.8 ± 10.8	37.4 ± 11.0	21	24	16 weeks	120 min	1 time/2 weeks	E-Health ME	Not mindfulness-based intervention	①②
Senra et al. 2025, (33)	Portugal	42.7 ± 7.7	42.5 ± 6.9	29	43	12 weeks	N/A	1 time/week	M-Health Audio-based exercises	None	①③

USA, The United States of America; EG, experimental group; CG, control group; the numbers “1” and “2” following the author’s name in Table 1 indicate two studies established in the same literature, respectively; ME, mindfulness-based eating exercises; ① BMI; ② Eating behaviour; ③ Binge eating.

Additionally, one three-arm study (36) with two eligible intervention arms, provided two effect sizes, one for emotional eating and one uncontrolled eating. Among the 10 studies, only four explicitly reported using intention-to-treat (ITT) analysis for their primary outcomes (29, 33, 34, 37), whereas the remaining six studies did not specify whether ITT was applied. Only three studies did not report the users’ engagement metrics (32, 35, 36).

Among the included studies, four delivered audio-based meditation as the primary activity (29–31, 33), two offered mindfulness-based eating exercises (32, 36), one provided video- or text-based exercises (35), one used breathing exercises (37), one provided a video meeting (16), and one did not specify the activity (34).

### 3.3 Risk of bias

**Figure 2:**
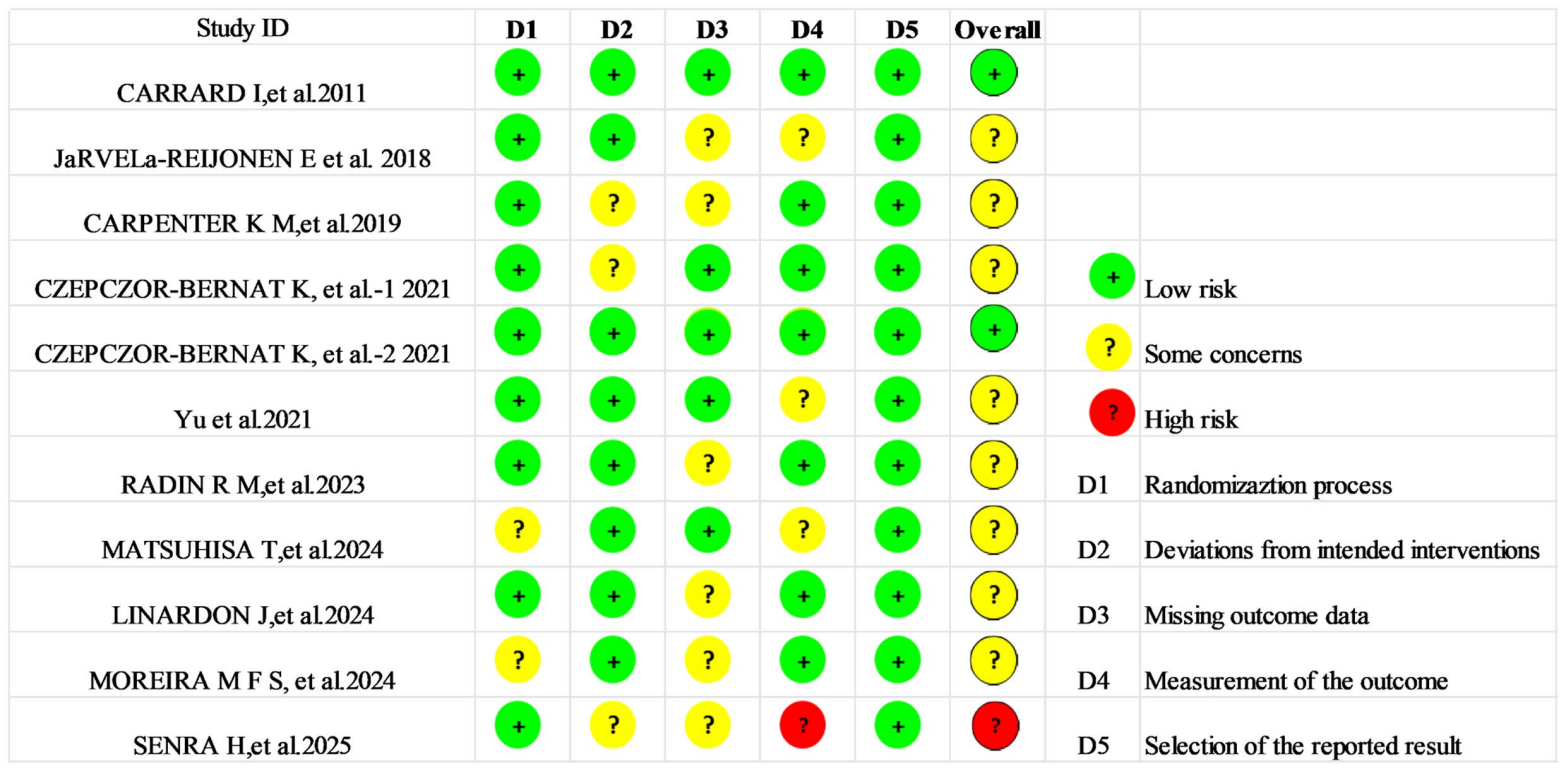
Risk of bias of included studies.

Figure 2 showed a summary of the risk-of-bias assessment results for the included studies. The majority (8 studies) adequately described randomization methods such as random number tables and computerized randomization. In terms of measurement bias, only one study was rated as having high risk in the outcome assessment process.

### 3.4 Meta-analysis results

#### 3.4.1 BMI

**Figure 3:**
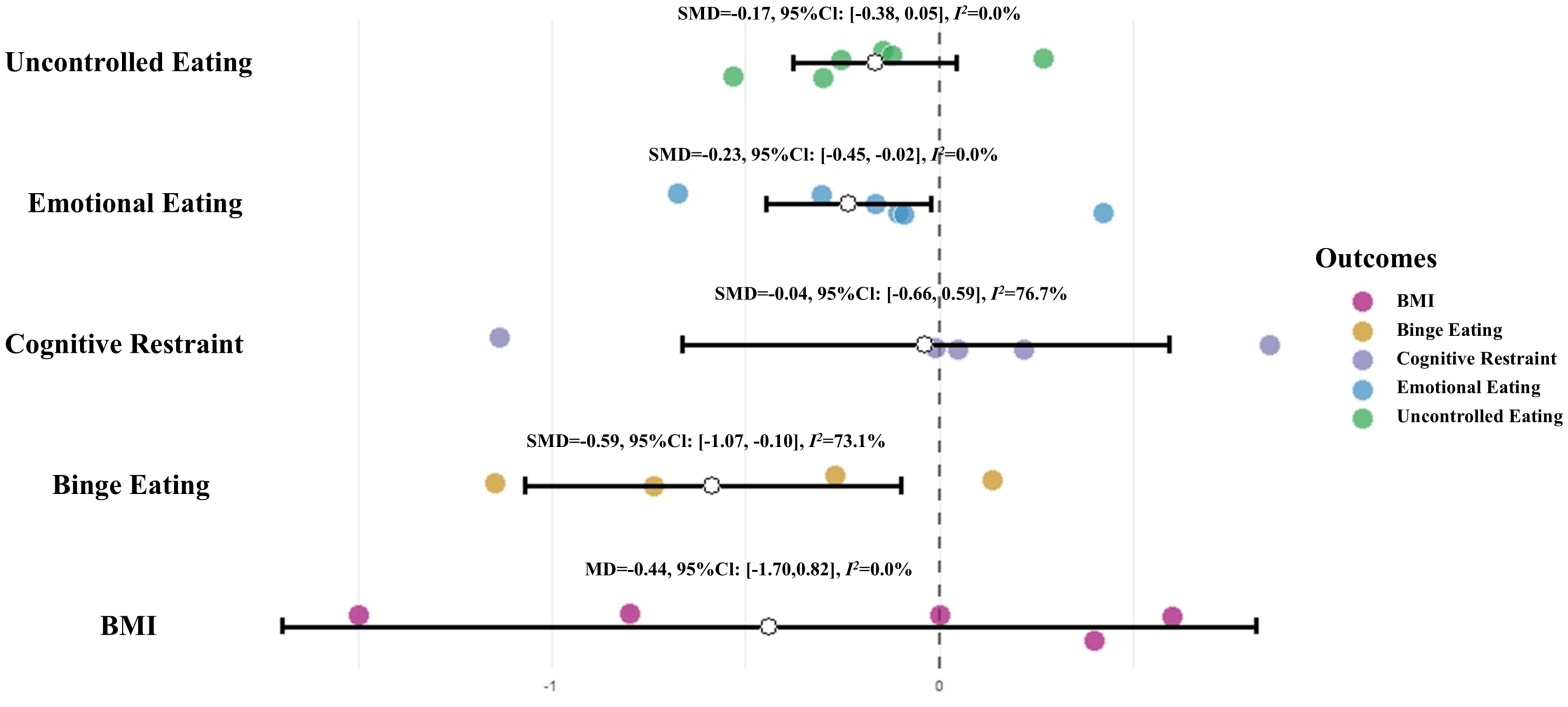
Forest plot.

Five studies (*k* = 5) reporting on BMI were enrolled in this meta-analysis. The pooled results revealed a non-significant reduction in BMI associated with the intervention [MD = −0.44, 95%CI: (−1.70, 0.82), *I*^2^ = 0.0%] (Figure 3).

#### 3.4.2 Binge eating

A meta-analysis of 4 studies (*k* = 4) on binge eating showed a significant, medium reduction associated with the intervention [SMD = −0.59, 95%CI: (−1.07, −0.10)], with considerable heterogeneity observed (*I*^2^ = 73.1%) (Figure 3).

#### 3.4.3 Cognitive restraint

The meta-analysis of cognitive restraint included 5 studies (*k* = 5). The pooled result was as follows: [SMD = −0.04, 95%CI: (−0.66, 0.59), *I*^2^ = 76.7%] (Figure 3). However, given the insufficient precision and the small number of studies, we refrained from drawing conclusion from these data. This outcome is therefore reported only descriptively, and the available evidence is insufficient to inform clinical interpretation.

#### 3.4.4 Emotional eating

The meta-analysis of emotional eating included 5 studies which containing 6 reports (*k* = 6). The pooled results revealed a small reduction associated with the intervention [SMD = −0.23, 95%CI: (−0.45, −0.02), *I*^2^ = 0.0%] (Figure 3).

#### 3.4.5 Uncontrolled eating

The meta-analysis of uncontrolled eating included 5 studies which containing 6 reports (*k* = 6). The pooled results revealed a non-significant reduction associated with the intervention [SMD = −0.17, 95%CI: (−0.38, 0.05), *I*^2^ = 0.0%] (Figure 3).

### 3.5 Subgroup analyses

Binge eating was analysed by intervention period in subgroup analyses. The results of the subgroup analysis are presented in Table 2.

**Table 2:** Subgroup analysis for Binge eating.

Subgroups	*k*	Effect size (95%CI)	*I*^2^ (%)	Test of heterogeneity		
				*Q*	df	*p*
Period (weeks)				0.12	1	0.73
>12	2	−0.47 (−1.26, 0.31)	41.2%			
≤12	2	−0.68 (−1.54, 0.18)	89.1%			

With regard to binge eating, the difference test between the “>12 w” subgroup (*k* = 2, SMD = −0.47, 95%CI: −1.26, 0.31) and the “≤12 w” subgroup (*k* = 2, SMD = −0.68, 95%CI: −1.54, 0.18) was not significant (*p* > 0.05), suggesting that these variables might not be the main modulating factors for the binge eating. Due to the limited studies, this finding should as the exploratory nature.

### 3.6 Sensitivity analyses

To verify the stability and reliability of the meta-analysis results, sensitivity analysis, excluding small-sample studies and those where mindfulness was only a minor component, revealed consistent effect size directions across all outcome indicators (Supplementary Tables S4 and S5). This finding suggested that despite considerable heterogeneity in certain outcomes, these studies did not substantially influence the overall meta-analysis conclusions. Collectively, the sensitivity analyses confirmed the robustness and stability of the meta-analysis findings.

In addition to the above exclusion, we tested the impact of the assumed pre-post correlation coefficient (r) on the pooled effect estimates. For BMI, binge eating, cognitive restraint, and uncontrolled eating, varying r from 0.30 to 0.70 did not change the direction or statistical significance of any pooled estimate, although larger r values produced slightly larger effect sizes. For emotional eating, the analysis under *r* = 0.3 yielded a non-significant pooled effect (SMD = −0.20, 95%CI: −0.41 to 0.01, *I*^2^ = 0%); whereas under *r* = 0.7, the pooled effect yielded significant (SMD = −0.30, 95%CI: −0.51 to −0.08, *I*^2^ = 24.8%). These findings indicated that the statistical significance of emotional eating is sensitive to the assumed pre-post correlation, therefore, this result should be interpreted with caution (Supplementary Tables S2 and S3).

Publication bias was evaluated using funnel plots, formal statistical testing for publication bias was supplemented by with Egger’s test when applicable. However, due to fewer than 10 studies were available for each outcome. Egger’s test was not performed, as minimum of 10 studies is generally required for robust bias assessment.

### 3.7 Certainty of evidence

Table 3 showed the results of the certainty of evidence (Supplementary Table S6).

**Table 3:** The certainty of evidence.

Outcomes	Certainty
BMI	⨁⨁ Low
Binge eating	⨁⨁ Low
Uncontrolled eating	⨁ Very low
Emotional eating	⨁⨁⨁ Moderate
Cognitive restraint	⨁⨁ Low

## 4 Discussion

This meta-analysis and systematic review was conducted to clarify the effectiveness of mindfulness-based digital health interventions on BMI and weight-related behaviours in adults. The results revealed no significant between-group differences in BMI. However, the interventions showed a trend toward significant benefits in reducing both binge eating and emotional eating compared with control groups.

### 4.1 BMI

Our findings on BMI are in line with those of Ruffault et al. (38), showing no significant effect. Similarly, Lyzwinski et al. (10) found no weight loss in their 11-week app MBIs among college students. By contrast, Jeffrey’s team discovered that MBIs significantly improved BMI (11).

This inconsistency may stem from: (1) The core components of MBIs include cultivating awareness, increasing attention to present experiences, fostering response flexibility, and enhancing emotional acceptance (39), rather than directly specified calorie expenditure or intake. (2) Unlike traditional approaches, this intervention may have a delayed effect on achieving significant BMI reduction, and longer follow-up period may be associated with lower BMI. (3) Although digital health platforms offer several advantages as the delivery modality, they may have certain shortcomings in terms of app design and user engagement. For example, if the design fails to attract users, they may struggle to maintain long-term use or may drop out, which could ultimately compromise intervention effectiveness (40, 41). (4) There are many factors influencing BMI, in addition to exercise, it may also be related to factors such as population characteristics, ethnicity (42), and smoking status (43), etc. These factors may dilute the impact of the intervention on the BMI.

### 4.2 Binge eating

This study found that mindfulness-based digital health interventions exert a medium effect on binge eating behaviour. A core maintenance factor of binge eating being is the tendency to cope with negative emotional energy deficits and avoid unpleasant experiences (44). Mindfulness may reduce binge eating by helping individuals become less controlled by specific emotions and thoughts. Traditional offline mindfulness interventions have demonstrated efficacy in clinical practice (45). For example, Kristeller et al. applied MBI to obese women with bulimia nervosa and achieved favorable clinical outcomes (5), a finding later corroborated by Daniela et al. (46). Building on this evidence, digital health interventions offer additional advantages, such as real-time feedback, repeatable practice, strong privacy protection, convenience, and cost-effectiveness (47), may further enhance efficacy by facilitating daily life and improving skill generalization.

However, the substantial heterogeneity observed across included studies indicates that the effect is not uniform. This may be attributable to variations in cultural settings (25, 26), age range, and intervention types. Consequently, the findings of this study are intended solely as a reference for clinicians; further and more in-depth research is needed to continue exploring this topic.

### 4.3 Eating behaviours

Within the framework of this study, mindfulness-based digital health interventions significantly improved emotional eating, but showed no significant effect on uncontrolled eating, and the available evidence is insufficient for cognitive restraint. These findings differ from those of K. Carriere et al. (48), who found greater efficacy of face-to-face MBI in improving overall eating behaviours. This suggested that digital MBI may yield weaker effects possibly because they are still in the early development and require further refinement of their protocols. Supporting this view, a systematic review by Manuela et al. (49) on digital health interventions for eating behaviour found no significant improvement, which may be explained by the fact that not all included digital interventions were mindfulness-based, and the participants were predominantly overweight and obese adults.

Regarding eating behaviour, improvements were primarily observed in emotional eating, whereas the effect on uncontrolled eating was not statistically significant. This finding aligns with Smith et al., who also found that MBIs effectively reduce emotional eating (50). However, Margarita et al. noted that MBIs not only significantly improve emotional eating but also have positive effects on other dimensions of eating behaviours (51). Similarly, Lyzwinski et al. (10) found slight but significant enhancements in both emotional eating and uncontrolled eating following a mindfulness application for intervention. The inconsistency between our results and previous findings may be partially attributed to the specific nature of emotional eating, which is triggered by negative emotional rather than physiological hunger (52), making it particularly responsive to MBIs. In addition, the positive outcomes in Lyzwinski et al. may have been facilitated by push notifications of meal times, which enhanced intervention adherence to some extent. Finally, our study restriction to mindfulness-based digital health interventions may reduce the depth of feedback and contextualized practice necessary to effectively modify complex behavioural constructs.

It is also worth noting that, while emotional eating improved significantly, BMI showed no significant change. This phenomenon may be explained by the fact that psychological and behavioural changes do not automatically translate into a sufficient caloric deficit, and that BMI typically requires a larger cumulative energy deficit to detect meaningful change (53). Carriere et al. found that MBIs had a moderate effect on weight loss, but a larger effect on obesity-related eating behaviours, suggesting that improvements in psychological and behavioural outcomes tent to be more pronounced than changes in body weight (48). Furthermore, emotional eating was primarily assessed using self-report questionnaires, which are sensitive to short-term changes, whereas BMI is not. Therefore, post-intervention assessments may have captured only early behavioural changes that had not yet had sufficient time to influence BMI. The clinical significance of a small effect size is debatable. A reduction of this magnitude may correspond to a modest improvement in eating behaviour that could support better weight management, but it is unlikely to produce rapid or dramatic changes. However, given the sensitivity analysis results, the beneficial effect on emotional eating should be interpreted with caution.

Regarding cognitive restraint, the available data are too limited to support any meaningful conclusion. Therefore, we refrain from interpreting this finding as a null finding and instead note that the current evidence does not permit reliable inference. Future studies with larger samples and more precise estimates are needed.

### 4.4 Limitations

Despite strict adherence to PRISMA guidelines, this study has several limitations: (1) The analyses of binge eating and cognitive restraint revealed substantial heterogeneity, potentially undermining the credibility of these findings. However, due to the limited information available and the uneven distribution of intervention characteristics across studies, we were only able to perform a subgroup analysis by intervention period, and this was conducted solely for binge eating. For cognitive restraint, a subgroup analysis could not be performed because the number of studies with different intervention periods was insufficient. (2) One included study had a very small-sample size. Although it can provide preliminary evidence, we retained it because it met our eligibility criteria; sensitivity analyses excluding this study did not materially change the direction or significance of the results. Nevertheless, readers should be aware of this limitation. (3) Although 7 of the 10 studies explicitly reported user engagement metrics, having access to the app does not mean that participants were actually practicing mindfulness. The lack of objective engagement data or a verification mechanism to confirm mindfulness practice, along with variations in digital literacy and technology access across age ranges and geographic settings, may have influenced intervention participation and outcomes. (4) The GRADE certainty of evidence ranged from very low to moderate across outcomes. (5) The absence of ITT reporting and potential attrition bias, may lead to overestimation or underestimation of the true intervention effects. Thus, future high-quality RCTs with larger sample sizes and standardized protocols are warranted.

## 5 Conclusion

We evaluated the effects of mindfulness-based digital health interventions on BMI and weight-related behaviours in adults. The interventions significantly improved emotional eating and binge eating but showed no clear effect on BMI. These findings suggested that future research should focus on continually refining the details of the intervention protocol to enhance study robustness.

## Data Availability

The original contributions presented in the study are included in the article/Supplementary material, further inquiries can be directed to the corresponding author.
