# Supplementary material for: Effects of mindfulness-based digital health interventions on BMI and weight-related behaviours in adults: a systematic review and meta-analysis
Source: Front Nutr. 2026 Jul 28;13:1874656. doi: 10.3389/fnut.2026.1874656 (PMC13457005; doi:10.3389/fnut.2026.1874656)
Supplement: Supplementary file 1 [file Table_1.DOCX]

Supplementary Material

# 1 Supplementary Figures and Tables

## 1.1 Supplementary Tables

**Table S1 Search Strategy**

| **PubMed** | | Results |
| --- | --- | --- |
| #1 | ("mindful eating"[Title/Abstract] OR "mindfulness-based eating"[Title/Abstract] OR "mindfulness-based intervention"[Title/Abstract] OR "mindfulness"[Title/Abstract]) | 18281 |
| #2 | ("digital"[Title/Abstract] OR "mobile"[Title/Abstract] OR "APP"[Title/Abstract] OR "application"[Title/Abstract] OR "smartphone"[Title/Abstract] OR "online"[Title/Abstract] OR "web-based"[Title/Abstract] OR "internet-based"[Title/Abstract] OR "e-health"[Title/Abstract] OR "m-health"[Title/Abstract] OR "digital health"[Title/Abstract]) | 1982901 |
| #3 | ("binge eating"[Title/Abstract] OR "binge-eating"[Title/Abstract] OR "loss of control eating"[Title/Abstract] OR "emotional eating"[Title/Abstract]) OR ("body weight"[Title/Abstract] OR "weight loss"[Title/Abstract] OR "BMI"[Title/Abstract] OR "body mass index"[Title/Abstract] OR “diet behaviour”[Title/Abstract] OR “eating behaviour”[Title/Abstract]) | 728883 |
| #4 | #1 AND #2 | 3626 |
| #5 | #3 AND #4 | 130 |
| **WOS** | |  |
| #1 | TI=(mindful eating OR mindfulness-based eating OR mindfulness-based intervention OR mindfulness) | 22316 |
| #2 | TI=(digital OR mobile OR app OR application OR smartphone OR online OR web-based OR internet-based OR m-health OR digital health) | 2420267 |
| #3 | TI=(binge eating OR binge-eating OR loss of control eating OR emotional eating OR body weight OR weight loss OR BMI OR body mass index OR diet behaviour OR eating behaviour) | 138561 |
| #4 | #1 AND #2 | 1073 |
| #5 | #3 AND #4 | 10 |
| **Scopus** | |  |
| #1 | TITLE-ABS-KEY(mindful eating OR mindfulness-based eating OR mindfulness-based intervention OR mindfulness) | 43949 |
| #2 | TITLE-ABS-KEY(digital OR mobile OR app OR application OR smartphone OR online OR web-based OR internet-based OR m-health OR digital health) | 13632978 |
| #3 | TITLE-ABS-KEY(binge eating OR binge-eating OR loss of control eating OR emotional eating OR body weight OR weight loss OR BMI OR body mass index OR diet behaviour OR eating behaviour) | 1368078 |
| #4 | #1 AND #2 AND #3 | 323 |

**Table S2.** Sensitivity analysis ( r = 0.3)

| Outcomes | k | Effect size (95%CI) | *I*² |
| --- | --- | --- | --- |
| BMI | 5 | -0.44 (-1.93, 1.05) | 0% |
| Binge eating | 4 | -0.50 (-0.91, -0.09) | 63.3% |
| Cognitive restraint | 5 | -0.02 (-0.57, 0.53) | 70.9% |
| Emotional eating | 6 | -0.20 (-0.41, 0.01) | 0% |
| Uncontrolled eating | 6 | -0.14 (-0.36, 0.07) | 0% |

**Table S3.** Sensitivity analysis ( r = 0.7)

| Outcomes | k | Effect size (95%CI) | *I*² |
| --- | --- | --- | --- |
| BMI | 5 | -0.44 (-1.42, 0.54) | 0% |
| Binge eating | 4 | -0.73 (-1.37, -0.10) | 83% |
| Cognitive restraint | 5 | -0.07 (-0.81, 0.68) | 82.9% |
| Emotional eating | 6 | -0.30 (-0.51, -0.08) | 24.8% |
| Uncontrolled eating | 6 | -0.21 (-0.42, 0.01) | 0% |

**Table S4.** Sensitivity analysis (omitting small-sample study)

| Outcomes | Effect size (95%CI) | *I*² |
| --- | --- | --- |
| BMI | -0.52 (-1.83, 0.80) | 0% |
| Binge eating | -0.49 (-0.70, -0.28) | 80.3% |
| Cognitive restraint | -0.06 (-0.30, 0.17) | 82.3% |
| Emotional eating | -0.25 (-0.47, -0.04) | 0% |
| Uncontrolled eating | -0.17 (-0.38, 0.05) | 0% |

**Table S5.** Sensitivity analysis (omitting studies in which mindfulness was merely a minor component)

| Outcomes | *k* | Effect size (95%CI) | *I*² |
| --- | --- | --- | --- |
| BMI | 3 | -0.42 (-1.94, 1.10) | 0% |
| Binge eating | 2 | -0.95 (-1.32, -0.58) | 15.2% |
| Cognitive restraint | 2 | -0.15 (-2.10, 1.80) | 93.6% |
| Emotional eating | 5 | -0.25 (-0.47, -0.04) | 0% |
| Uncontrolled eating | 5 | -0.17 (-0.38, 0.05) | 0% |

**Table S6 GRADE**

| **Certainty assessment** | | | | | | | **№ of patients** | | **Certainty** | |
| --- | --- | --- | --- | --- | --- | --- | --- | --- | --- | --- |
| **№ of studies** | **Study design** | **Risk of bias** | **Inconsistency** | **Indirectness** | **Imprecision** | **Other considerations** | **[intervention]** | **[comparison]** | **Absolute (95% CI)** |  |
| **BMI** | | | | | | | | | | |
| 5 | randomised trials | serious^a^ | not serious | not serious | serious^b^ | none | 124 | 145 | MD **0.44 lower** (1.7 lower to 0.82 higher) | ⨁⨁◯◯ Low^a,b^ |
| **Binge eating** | | | | | | | | | | |
| 4 | randomised trials | serious^a^ | serious^c^ | not serious | not serious | none | 196 | 190 | SMD **0.59 SD lower** (1.07 lower to 0.1 lower) | ⨁⨁◯◯ Low^a,c^ |
| **Uncontrolled eating** | | | | | | | | | | |
| 5 | randomised trials | serious^a^ | serious^c^ | not serious | serious^b^ | none | 156 | 154 | SMD **0.17 SD lower** (0.38 lower to 0.05 higher) | ⨁◯◯◯ Very low^a,b,c^ |
| **Emotional eating** | | | | | | | | | | |
| 5 | randomised trials | serious^a^ | not serious | not serious | not serious | none | 208 | 157 | SMD **0.23 SD lower** (0.45 lower to 0.02 lower) | ⨁⨁⨁◯ Moderate^a^ |
| **Cognitive restraint** | | | | | | | | | | |
| 5 | randomised trials | serious^a^ | not serious | not serious | serious^b^ | none | 156 | 154 | SMD **0.04 SD lower** (0.66 lower to 0.59 higher) | ⨁⨁◯◯ Low^a,b^ |

**CI:** confidence interval; **MD:** mean difference; **SMD:** standardised mean difference

#### Explanations

a. Most of the included studies were rated as "some concerns" on the RoB2 tool

b. The 95% confidence interval crossed zero, and the sample size was small.

c. High heterogeneity

## 1.2 Supplementary Figures

**Figure S1.Forest plot of BMI**

**Figure S2.Forest plot of binge eating**

**Figure S3.Forest plot of cognitive restraint**

**Figure S4.Forest plot of emotional eating**

**Figure S5.Forest plot of uncontrolled eating**
